# End-Stage Renal Disease in a Patient With Schmidt’s Syndrome: A Case Report

**DOI:** 10.7759/cureus.27342

**Published:** 2022-07-27

**Authors:** Hamza Ismaeel, Shahan Tariq, Zohaib Akram

**Affiliations:** 1 Internal Medicine, Rawalpindi Medical University, Rawalpindi, PAK; 2 Internal Medicine, Pak Emirates Military Hospital, Rawalpindi, PAK; 3 Internal Medicine, Mayo Hospital, Lahore, PAK

**Keywords:** addison's disease, endocrinology, schmidt syndrome, polyendocrine autoimmune syndromes, end-stage renal disease (esrd)

## Abstract

Schmidt's syndrome constitutes Addison's disease in conjunction with autoimmune hypothyroidism or type 1 diabetes mellitus. It has misleading symptomology and an unclear order of presentation of symptoms. This often results in missed and late diagnosis. Chronic kidney disease is a rarely reported phenomenon in Schmidt’s syndrome. Multiple factors may have the potential to cause renal failure, such as Addison's disease and/or hypothyroidism, the understanding of which is still evolving. A 45-year-old gentleman who is a known case of Schmidt's syndrome presented to us with fatigue, anorexia, and weight gain. Further evaluation revealed a picture of chronic kidney disease. We would like to alert fellow peers of this potential complication and the importance of screening as well as timely diagnosis.

## Introduction

Polyendocrine autoimmune syndromes (PAS) are characterized by a host of manifestations affecting the endocrine and non-endocrine systems. They are classified into two major subtypes: type 1 and type 2. Schmidt's syndrome or PAS 2 is defined as having Addison’s disease in addition to autoimmune hypothyroidism and autoimmune diabetes mellitus type 1 or both. It often presents in a nonspecific order with complex symptomology; making the diagnosis a cumbersome task. A combination of Addison’s disease with hypothyroidism has been found to occur more frequently [1]. The prevalence of PAS 2 has been estimated at 1.4-2.0 per 100,000, most commonly occurring at the age of 30-40 years with women being affected three times more than men [2]. Furthermore, the manifestation of chronic kidney disease (CKD) is unusual and rare in Schmidt’s syndrome [3].

## Case presentation

A 45-year-old male with Schmidt’s syndrome presented with complaints of generalized weakness, abdominal pain, and weight gain. Around 15 days ago, the patient started to feel fatigued and weak while performing everyday activities. He also complained of nausea, anorexia, and pruritus. The patient reported an unintentional weight gain of 11 kilograms for the past six months. He had been trying to lose weight with a regimen of diet and exercise but to no avail. In addition, generalized abdominal pain started three days ago. It was episodic, colicky in character, non-radiating, 2/10 in intensity, and associated with abdominal distension. The patient denied burning sensations, diarrhea, eating out, change in stool color, night sweats, fever, or use of alcohol or tobacco. No history of allergy to medication and food could be obtained.

Past medical history revealed that the patient was a known case of hypothyroidism and Addison's disease for the past 17 years. He was subsequently managed with thyroxine and glucocorticoids. On further inquiry, a history of multiple acute kidney injury (AKI) episodes over the past several years were reported, the most recent one occurring a year ago. He had been irregular on follow-up.

**Figure 1:**
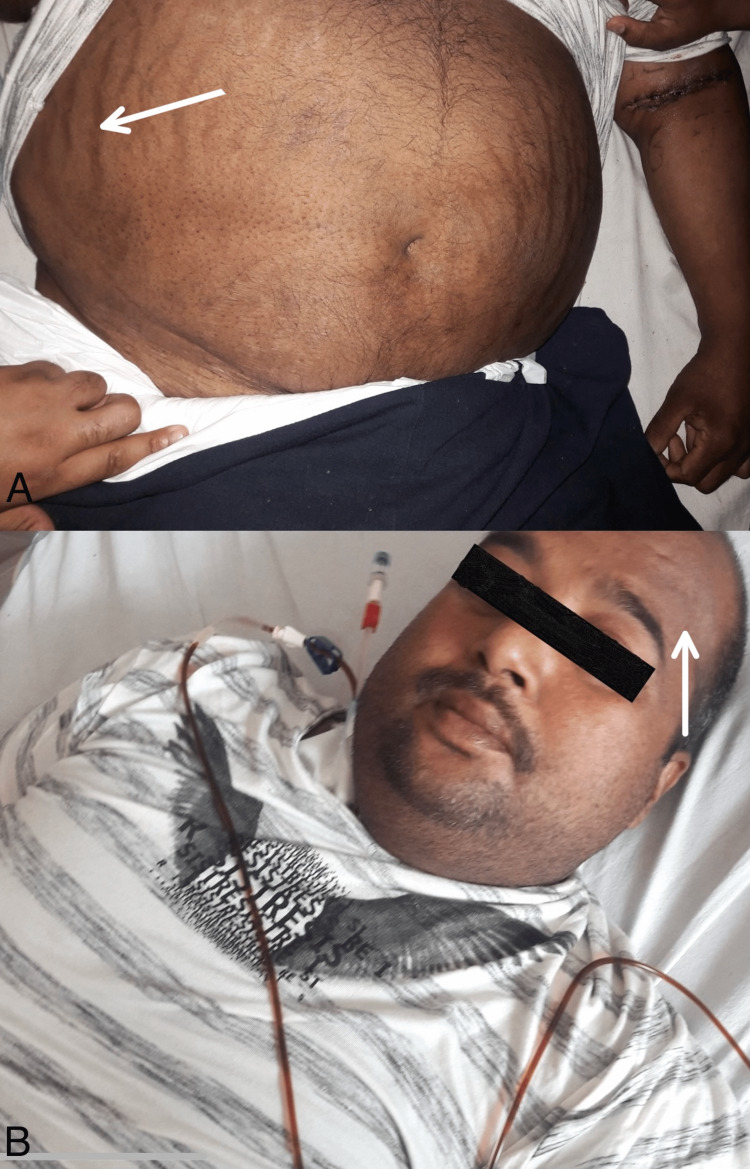
A) Image showing abdominal distention, striations (arrow), and hyperpigmentation; B) Patch of hyperpigmentation visible on the patient's forehead

Examination demonstrated an alert and oriented middle-aged individual. All vital signs were within the normal range. On inspection of the abdomen, visible distention, striations, and hyperpigmentation were observed (Figure 1). Palpation demonstrated a soft, non-tender abdomen. A non-resonant note was appreciated on percussion. Normal bowel sounds were heard on auscultation.

**Figure 2:**
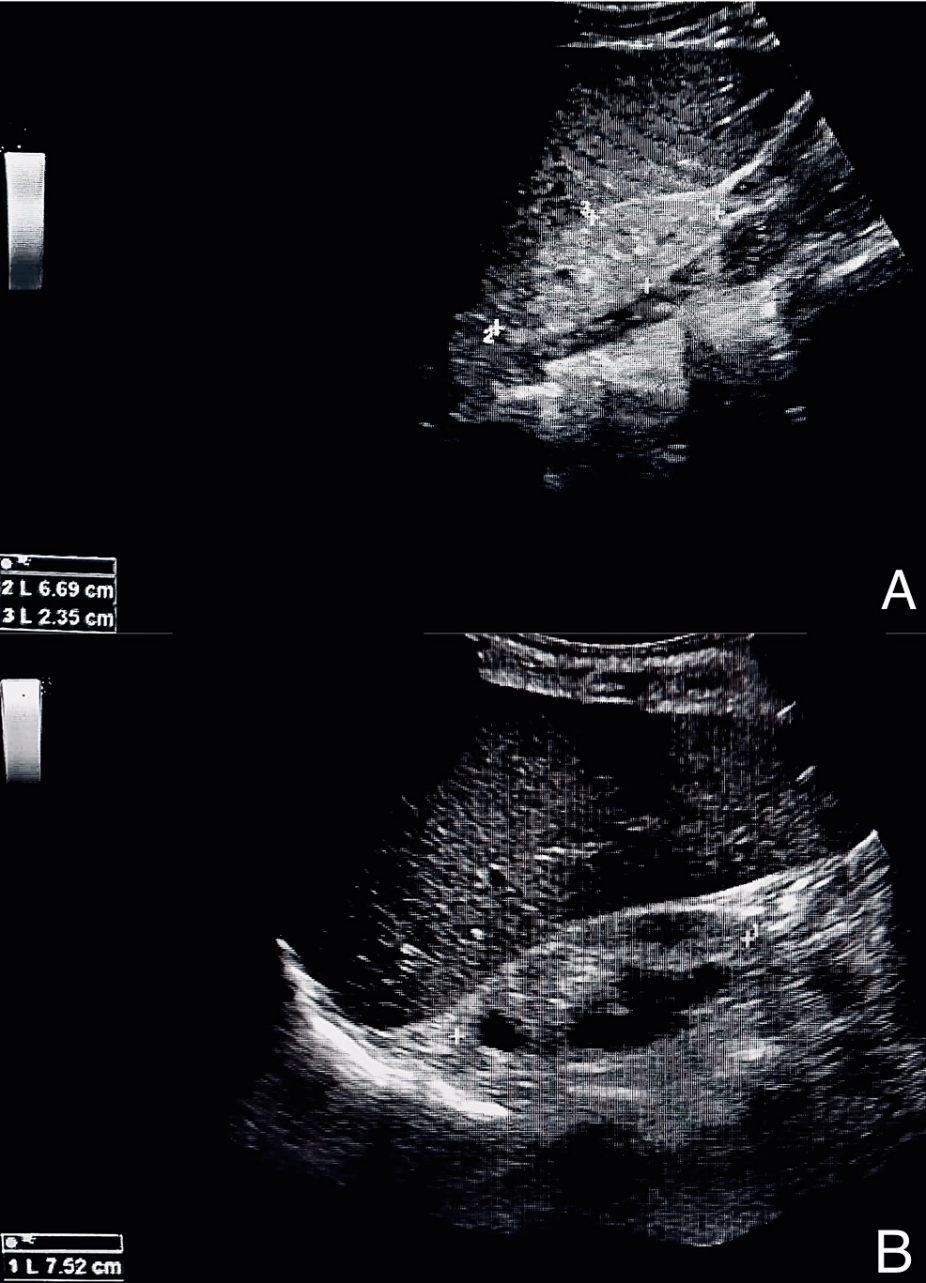
Bilateral shrunken kidneys (sizes 6.69 and 7.52 cm, respectively)

Complete blood count (CBC), renal function tests (RFTs), liver function tests (LFTs), serum electrolytes, arterial blood gases (ABGs), urinalysis, and parathyroid hormone levels (PTH) were ordered. RFTs exhibited a high level of serum creatinine at 10.2 mg/dl. The estimated glomerular filtration rate (GFR) was 3 ml/min/1.73 m² (CKD stage 5/end-stage renal disease (ESRD)). Elevated levels of serum potassium 6.8 mg/dl and Phosphate; 8.6 mg/dl were observed. PTH levels were markedly raised to 553 pg/dl. Urinalysis revealed red blood cells, glucosuria, and significant proteinuria. Hematocrit of 20.6%, mean corpuscular volume of 77.7 fL, hemoglobin of 6.8 g/dl, and red blood cell count of 2.65 million/ml were noted. Arterial blood gases revealed metabolic acidosis with a pH of 7.196, partial pressure of carbon dioxide (PCO2) of 8.9 mmHg, and bicarbonate 3.4 mmol/L. LFTs were normal. I/V calcium gluconate was immediately administered due to high potassium levels. Ultrasonography findings revealed bilateral shrunken kidneys (Figure 2). Due to this symptom complex, nephrology was consulted. After evaluation, the patient was started on hemodialysis. Serial monitoring of electrolytes and arterial blood gases was done. A central line was passed, and he was put on strict fluid input/output monitoring. Over the course of his stay in the hospital, at times, his blood pressure readings revealed mild hypotension. Further complications arose when he developed sepsis accompanied by erratic blood glucose levels. Broad-spectrum antibiotics along with I/V paracetamol were initiated. Unfortunately, the patient passed away as a result of these complications.

## Discussion

Fifty percent (50%) of cases with Addison’s disease will develop some type of autoimmune disease later in life [2,4]. Moreover, around 25% of patients afflicted with an autoimmune syndrome will develop another one as well [2]. Addison’s disease is often misdiagnosed as CKD due to a host of common findings, and clinicians have stressed the need to consider ESRD as a potential complication in the past in such patients [5-6]. Our understanding of renal failure in Schmidt's syndrome is still evolving. Prognosis in Schmidt's syndrome is plagued by missed or delayed diagnosis and poor survival [7]. Furthermore, hypothyroidism has been reported to decrease renal function and increase the risk of CKD in patients [8-10]. Reports suggest that it can precipitate native CKD to AKI as well [9]. Our patient also presented to us last year with acute renal failure. Studies are warranted to rule it out as a potential culprit of acute renal failure and ESRD in Schmidt's syndrome. Screening for diabetes and hypothyroidism must be done every five years; this has been emphasized in various studies [9,11-12]. CKD in Schmidt’s syndrome is a misunderstood phenomenon; therefore, it must be investigated. We would like to highlight ESRD as a rare complication in Schmidt's syndrome. Thus, patient education, screening, and early diagnosis are of invaluable importance.

## Conclusions

To sum up, the purpose of this report is to highlight vital caveats. Our understanding of chronic kidney disease in Schmidt's syndrome is limited, which must be investigated further. Varied symptomology in Schmidt's syndrome continues to baffle physicians. This results in both missed/late diagnosis and underestimation of its true potential in wreaking havoc on patients. Poor survival after diagnosis is of utmost concern. Addison's disease is often misdiagnosed as renal failure. It has been suggested that hypothyroidism plays a role in renal failure as well. This association also needs to be further elaborated. Therefore, clinical trials have become the need of the hour. Our patient rapidly deteriorated into end-stage renal disease and did not survive. Timely diagnosis and intervention are key. Screening along with regular follow-up for hypothyroidism and diabetes is also of prime importance in such cases.
